# High-altitude Cerebral Edema and High-altitude Pulmonary Edema Diagnosed in the Desert: A Case Report

**DOI:** 10.5811/cpcem.3851

**Published:** 2024-05-28

**Authors:** Bryn Walsh, Suneil Agrawal

**Affiliations:** Desert Regional Medical Center, Palm Springs, California

**Keywords:** acute mountain sickness, high altitude pulmonary edema, high altitude cerebral edema, case report, computed tomography

## Abstract

**Introduction:**

Acute mountain sickness, high-altitude pulmonary edema (HAPE), and high-altitude cerebral edema (HACE) are a spectrum of high-altitude conditions, with HACE being the most life-threatening. Most cases develop at altitudes of greater than 4,000 meters (∼13,000 feet) above sea level and after one to five days.

**Case Report:**

A previously healthy 46-year-old female presented to the emergency department with ataxia, altered mental status, and vomiting that developed after rapidly ascending to ∼2,400 meters (∼7,800 feet) above sea level. She was treated for HACE and HAPE with resolution of her symptoms within 24 hours.

**Conclusion:**

High-altitude pulmonary edema and HACE can develop rapidly and at moderate altitudes. Expeditious recognition and treatment is imperative to avoid life-threatening complications.

CPC-EM Capsule What do we already know about this clinical entity?

*Most cases of high altitude cerebral edema (HACE) and high altitude pulmonary edema (HAPE) occur at greater than >4000 m (∼13000 ft) above sea level.*

What makes this presentation of disease reportable?

*This case highlights a patient who developed symptoms of HACE and HAPE within hours of rapidly ascending to ∼2400 m (∼7800 ft) above sea level.*

What is the major learning point?

*HAPE and HACE can occur more rapidly and at lower elevations than previously thought.*

How might this improve emergency medicine practice?

*Given the high mortality rate in delayed diagnosis of HAPE and HACE, it is imperative to keep a high index of suspicion when susceptible individuals present.*

## INTRODUCTION

High-altitude conditions involve a spectrum of diseases including acute mountain sickness (AMS), high-altitude pulmonary edema (HAPE), and high-altitude cerebral edema (HACE).^1^ ^–^ ^3^ Acute mountain sickness manifests with non-specific symptoms, most commonly headache, nausea, and fatigue. It usually develops 2–12 hours after initial arrival at high elevation.^1^ High-altitude pulmonary edema can present with exertional dyspnea, chest tightness, cough, and decreased exercise capacity.^4^ High-altitude cerebral edema generally presents with varying levels of confusion, ataxia, altered mental status, and vomiting, with ataxia present in up to 60% of reported cases.^1^ ^,^ ^4^ ^,^ ^5^ Early HACE may manifest as social withdrawal and drowsiness.^4^ High-altitude cerebral edema represents the least common form of high-altitude illness; however, it is the most important to diagnose and treat as the condition can rapidly progress to coma secondary to brain herniation and death in as few as 12 hours.^1^ ^,^ ^6^

The reported prevalence of HACE between 4,200–5,000 meters (m) (∼13,800 to ∼16,400 feet [ft]) above sea level is 0.5–1%.^3^ ^,^ ^4^ When HACE does present, it is thought to occur within 1–5 days of an ascent greater than 2,500–3,000 m (8,200–9,800 ft) above sea level, and it is rarely seen at altitudes lower than 2,500 m (8,200 ft) above sea level or within the first 24 hours of arrival.^4^ The most important factors in the development of symptoms are the rate of ascent and period of acclimatization.^1^ This case highlights a patient who developed symptoms of HACE and HAPE within hours of rapidly ascending to ∼2,400 m (∼7,800 ft) above sea level, which only few cases have previously demonstrated.

## CASE REPORT

A 46-year-old female with no past medical history and taking no medications presented to the emergency department (ED) via emergency medical services (EMS) for acute altered mental status that developed while hiking on Mount San Jacinto in Southern California. The patient had traveled from the coastal region of Southern California (∼20 m [∼60 ft] above sea level) to Palm Springs, CA, (∼150 m [∼490 ft]) above sea level with a wilderness group. After ascending via the Palm Springs Aerial Tramway over a 10–12 minute period and arriving at the base camp station (∼2,400 m [∼7,800 ft] above sea level), the patient began complaining of worsening malaise, nausea, chest tightness, and headache. Her symptoms worsened while hiking a short distance to a campsite (∼2,800 m [∼9,200 ft]). Shortly after arriving, the patient began vomiting, which prompted the team leader to descend on the tram with the patient and call EMS. During descent, the patient became more confused and ataxic, requiring assistance with ambulation. On EMS arrival at the base of the tram, the patient was markedly altered and agitated.

In the ED, vitals were blood pressure 132/86 millimeters of mercury, heart rate 84 beats per minute, respirations 21 breaths per minute, and oxygen saturation 100% on 2 liters nasal cannula. On physical exam, the patient was noted to be tachypneic, mumbling incomprehensible sounds, only opening eyes to voice, and withdrawing to pain. She was not answering questions or following commands. She was intermittently combative and fighting to get out of bed. Her Glasgow Coma Score was 10 (eyes-3, voice-2, motor-5). There was no evidence of trauma.

Workup in the ED included electrocardiogram, complete blood count, metabolic panel, urinalysis, drug screen, ethanol level, acetaminophen level, salicylate level, coagulation studies, quantitative human chorionic gonadotropin, chest radiograph (CXR), and computed tomography (CT) of the head. The only lab abnormalities were in the metabolic panel, with a sodium of 128 milliequivalents per liter (mEq/L) (reference range: 135–145 mEq/L), potassium of 3.1 mEq/L (3.5–5.0 mEq/L), carbon dioxide of 18 millimoles (mmol)/L (20–32 mmol/L), and elevated anion gap at 22 mEq/L (7–13 mEq/L). Ethanol, acetaminophen, salicylate, and urine drug screen were negative. The CXR (Image) showed diffuse pulmonary vascular congestion. She remained quite agitated in the ED, requiring 3 milligrams (mg) of lorazepam intravenously (IV). The head CT interpretation was limited due to patient motion because of agitation, although no acute intracranial abnormality was seen.

**Image.**
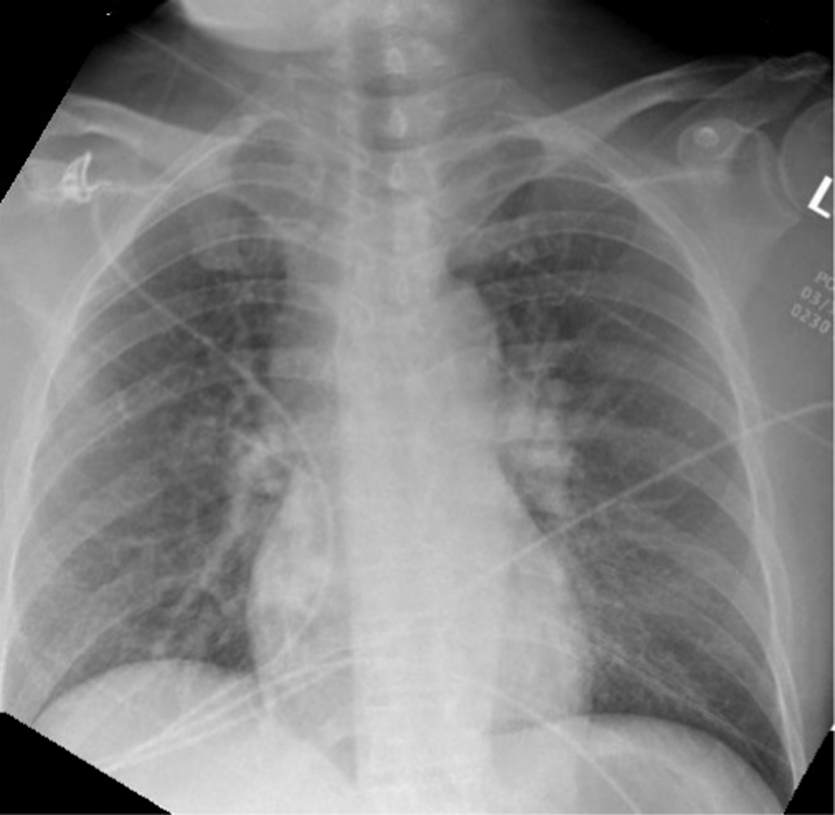
One view anterior-posterior chest radiograph obtained due to patient’s complaint of chest tightness during ascent to high altitude and tachypnea on presentation. Pulmonary vascular congestion is seen throughout. There is no evidence of pneumothorax, cardiomegaly, or consolidation.

Without another obvious cause of her hypoxia and altered mental status, HACE/HAPE treatment was started in the ED. She was given 250 mg acetazolamide IV and 8 mg dexamethasone IV. The patient was admitted to the hospital for observation, with neurology and nephrology specialist consultation for altered mental status and minor electrolyte abnormalities. Her symptoms completely resolved the following day, and her lab values normalized. Her mild hyponatremia of 128 mmol/L was not thought to be the main driving factor behind her symptoms. At the time of discharge she had no memory of descent, EMS transport, or her time in the ED. Once awake, she denied any previous drug or alcohol use.

## DISCUSSION

In the progression of high-altitude disease, AMS generally develops first.^5^ Symptoms are usually seen after 2–12 hours and reach maximum intensity at 18–24 hours at altitudes greater than 2,500 m (∼8,200 ft) above sea level.^1^ ^,^ ^4^ ^,^ ^7^ High-altitude cerebral edema and HAPE are rare, and usually occur at higher altitudes (>4,000 m [>13,000 ft]) after a longer period of time (1–5 days).^4^ Progression of AMS to HACE is thought to result from disruption of the blood brain barrier, intracellular edema and, possibly, venous outflow obstruction.^2^ ^,^ ^5^ Hypoxia leads to cerebral vasodilation and increased capillary pressure, eventually leading to fluid shifts into cells and cerebral edema.^2^ ^,^ ^5^ Hypoxia and cerebral vasodilation is thought to cause the initial headaches and lethargy seen in AMS, and alert patients can rapidly deteriorate to coma (within 12 hours), although this is rare and usually occurs at extreme altitudes.^2^ ^,^ ^5^ ^,^ ^8^ In HAPE, pulmonary artery pressures increase from hypoxic pulmonary vasoconstriction, causing capillary hypertension and fluid shifts.^4^ ^,^ ^5^ Of patients with HAPE, 15% will also have HACE, and HACE has been reported at altitudes as low as 2,500 m (∼8,200 ft) above sea level in patients who have concomitant HAPE.^1^ ^,^ ^7^ ^,^ ^9^

Risk factors for increased susceptibility for high-altitude illnesses include female gender, younger age, and a history of migraines.^3^ In the case described above, the patient presented with classic symptoms of both HACE (ataxia, altered mental status, vomiting) and HAPE (chest tightness, hypoxia, tachypnea). It is possible that our patient’s gender potentially contributed to her rapid progression. At 46 years of age, she was on the cusp of the defined age risk of AMS, which may have also played a part. The patient had no reported history of migraines. History of AMS has also been cited as a risk factor for recurrent episodes; however, she had no reported history of AMS.^3^

Our patient’s initial head CT, although limited secondary to motion artifact, did not reveal any obvious edema. When findings are present, CT of the head can show diffuse low density in the entire cerebrum, white matter signal attenuation with flat gyri and effaced sulci, and small ventricles.^5^ ^,^ ^8^ ^,^ ^10^ Magnetic resonance imaging (MRI) findings can include edema in the genu and splenium of the corpus callosum and the subcortical white matter.^1^ ^,^ ^5^ Microbleeds have also been seen in the cerebral white matter tracts of HACE survivors, which were not seen in patients with AMS or HAPE only.^1^ ^,^ ^7^ ^,^ ^11^ Cerebral white matter cytotoxic edema on brain MRI is more consistently seen after 22 hours of symptom onset.^1^ Hackett in 1998 detailed nine patients with HACE, all of whom recovered with treatment. Four patients who were defined as “moderately ill” had normal MRIs.^11^ In a recent study conducted by Long et al, the brain CT and MRI from 30 patients diagnosed with HACE were reviewed from January 2012–August 2022. Findings revealed a 100% sensitivity and 100% specificity of MRI diagnosis, and a 23.3% sensitivity and 100% specificity of CT diagnosis.^12^ Unfortunately, brain MRI was not done on our patient during hospitalization. Given the broad potential findings on CT head with its poor sensitivity, if clinical suspicion is high enough treatment should not be delayed due to negative CT head imaging.

High-altitude pulmonary edema can manifest as patchy lung infiltrates on CXR, which are usually unevenly distributed.^4^ ^,^ ^5^ ^,^ ^13^ As in HACE, CXR findings resolve as clinical features improve.^13^ While our patient’s CXR showed pulmonary edema consistent with HAPE, the distribution was more even than what is classically seen. Lower respiratory tract infections before travel are a risk factor that may account for HAPE at low altitudes.^4^ Viruses could alter the permeability of the alveolar-capillary barrier and lower the pressure required for formation of edema.^4^ To our knowledge, the patient had no known viral symptoms before ascent. The case also occurred before the 2019 coronavirus pandemic.

Once there is suspicion for a high-altitude condition of any degree, the most important treatment is descent. This is especially true in individuals with HACE due to the cerebral edema and risk of brain herniation.^2^ ^,^ ^12^ Wilderness Medical Society Guidelines recommend that rather than treating patients based on altitude only, treatment should be based on symptoms.^9^ Symptoms usually resolve after descent of 300–1,000 m (∼980–3,200 ft) above sea level.^9^ Interestingly, our patient’s condition worsened with descent. Once descended or if descent is not an option, low-flow oxygen can be applied with an oxygen saturation goal of greater than 90% for patients with suspected HAPE. The use of dexamethasone in the treatment of AMS and HACE is widely accepted, with a recommended dose of 8 mg (intramuscularly, IV, or orally), followed by 4 mg every six hours until symptoms resolve.^2^ ^,^ ^5^ ^,^ ^9^ Dexamethasone may help with HAPE, although this is unclear.^9^ There is limited data on the use of acetazolamide as a treatment for AMS or HACE. However, current literature recommends consideration of its use in AMS.^12^ For HAPE, acetazolamide may cause hypotension, especially if there is concomitant dehydration, which can worsen dyspnea.^9^ Loop diuretics should be avoided in HACE due to associated dehydration.^5^ ^,^ ^9^

## CONCLUSION

Acute mountain sickness, HAPE, and HACE are a spectrum of conditions under the umbrella of high-altitude illnesses. While rarely seen, and generally only at very high elevations (>4,000 m [>13,000 ft] above sea level), HAPE and HACE can occur more rapidly and at lower elevations than previously thought. Given the high mortality rate in delayed diagnosis of HAPE and HACE, it is imperative that physicians keep a high index of suspicion when potentially susceptible individuals present. Once high-altitude sickness is diagnosed, prompt treatment is crucial to avoid life-threatening complications.
